# Recipe for High Moment Materials with Rare-earth and *3d* Transition Metal Composites

**DOI:** 10.1038/srep29307

**Published:** 2016-07-06

**Authors:** Carmine Autieri, P. Anil Kumar, Dirk Walecki, Samira Webers, Mark A. Gubbins, Heiko Wende, Biplab Sanyal

**Affiliations:** 1 Department of Physics and Astronomy, Uppsala University, Box-516, 75120 Uppsala, Sweden; 2 Faculty of Physics Duisburg-Essen (CeNIDE), University of Duisburg-Essen, D-47057 Duisburg, Germany; 3 Seagate Technology, 1 Disc Drive, Springtown, Northern Ireland BT48 0BF, United Kingdom

## Abstract

Materials with high volume magnetization are perpetually needed for the generation of sufficiently large magnetic fields by writer pole of magnetic hard disks, especially for achieving increased areal density in storage media. In search of suitable materials combinations for this purpose, we have employed density functional theory to predict the magnetic coupling between iron and gadolinium layers separated by one to several monolayers of *3d* transition metals (Sc-Zn). We demonstrate that it is possible to find ferromagnetic coupling for many of them and in particular for the early transition metals giving rise to high moment. Cr and Mn are the only elements able to produce a significant ferromagnetic coupling for thicker spacer layers. We also present experimental results on two trilayer systems Fe/Sc/Gd and Fe/Mn/Gd. From the experiments, we confirm a ferromagnetic coupling between Fe and Gd across a 3 monolayers Sc spacer or a Mn spacer thicker than 1 monolayer. In addition, we observe a peculiar dependence of Fe/Gd magnetic coupling on the Mn spacer thickness.

High magnetic moment materials are used in several technological applications such as write heads in hard disks, generators, transformers, electrical vehicles and wind turbines^1^. Many different ferromagnetic materials with different characteristic features were created but the largest room temperature saturation magnetization (*M*_*S*_) remains unbeaten by the Slater-Pauling maximum (SPM) of the FeCo magnetization curve^2^,^3^. The Slater-Pauling curve is the plot of the magnetic moments versus doping in FeCo alloy and it reaches its maximum value *μ*_0_*M*_*S*_ = 2.45 T for Fe_65_Co_35_ composition. Other materials were proposed to exceed the Slater-Pauling limit but no definitive conclusion was made about reaching a saturation moment at room temperature larger than the SPM^4^.

The ferromagnetic *4f* rare earth metals like Gd exhibit, far below their Curie temperatures, higher volume magnetization than the limit set by SPM. However, we need to increase the low Curie temperature of these materials to make them usable for room temperature applications. In the past, multilayers composed of Fe and Gd have been studied but it is well known that magnetic *3d* elements couple antiferromagnetically to *4f* elements resulting in a reduced magnetic moment of the heterostructures. The interface between different materials offers the chance to manipulate the interplay between magnetic, orbital, electronic and lattice degrees of freedom giving rise to behavior that can differ from that of the bulk. In order to realize a material with a combination of both high spin magnetic moment and high Curie temperature, heterostructures of rare-earth-metal (e.g., Gd) and Fe with a high Curie temperature^5^ intermediated by a single Cr layer was proposed theoretically and a proof of principle experimental verification was also presented. However, it was also shown in the same work that only a few of Gd layers get coupled to the underlying Fe. In this work we extend that study by considering the whole series of *3d* transition metals ranging from Sc to Zn. For this purpose we use the Fe/X/Gd heterostructure with X *3d* transition metal as spacer between Fe and Gd. Fe and Gd will still be considered due to their high Curie temperature and high magnetic moment respectively. Adopting the same geometrical mode as reported in ref. 5, we will demonstrate that it is possible to create heterostructures with ferromagnetic coupling between Fe and Gd via other *3d* transition metal spacers too. A very large energy difference between ferromagnetic and antiferromagnetic configuration is a clear indication that this coupling can persist also at high temperature. It is important to note that the overcoming of the SPM by Fe/X/Gd heterostructures proposed here is by the way of enhancing the magnetic ordering temperature of Gd and is not due to the enhancement of the moments of either Fe or Gd.

Among the *3d* transition metals Fe, Co and Ni are ferromagnetic (FM) while Cr and Mn are antiferromagnetic (AFM) in their bulk form. The rest of the transition metals that we study as spacer layers are non-magnetic. At the interface between FM and non-magnetic (NM) materials, two phenomena are observed from magnetic point of view. One is the reduction of the local magnetic moment at the interface of the FM phase^5^,^6^ and the other is the induced magnetic moment in the NM interface layer. The induced magnetic moment can be parallel or anti-parallel to the magnetic moment of the FM phase depending on the charge transfer^7^. In case of one monolayer spacer, Ni and Co layers couple ferromagnetically to the Fe layer but antiferromagnetically to the Gd layer, while Cr and Mn couple antiferromagnetically to both the Fe and Gd layers. For the AFM spacer layers, with increasing the number of layers, an oscillating behavior of the magnetic coupling was found while the situation does not change for Ni and Co interlayers. For the NM interlayer an oscillating behaviour was found too increasing the number of interlayer, the oscillatory nature is consistent with the predictions of Ruderman, Kittel, Kasuya and Yosida (RKKY) theory^8^,^9^,^10^ but the strength of the interaction decays quite rapidly after few monolayers. While the RKKY oscillation is a long range phenomenon, the oscillation found in these heterostructures are short range.

The theoretical predictions are compared to experimental magnetic configurations in two trilayer systems; one with a non-magnetic Sc spacer and the other with magnetic Mn spacer. Macroscopic magnetic data confirms the breaking of antiferromagnetic coupling between Fe and Gd on insertion of Sc or Mn spacer in between. It is also observed that the magnetic coupling between Fe and Gd persists to thicker Mn spacer layer while the coupling is diminished for a Sc spacer above 3 monolayers (ML) thick.

## Results and Discussion

### Ab-initio study of the single interlayer

**Figure 1:**
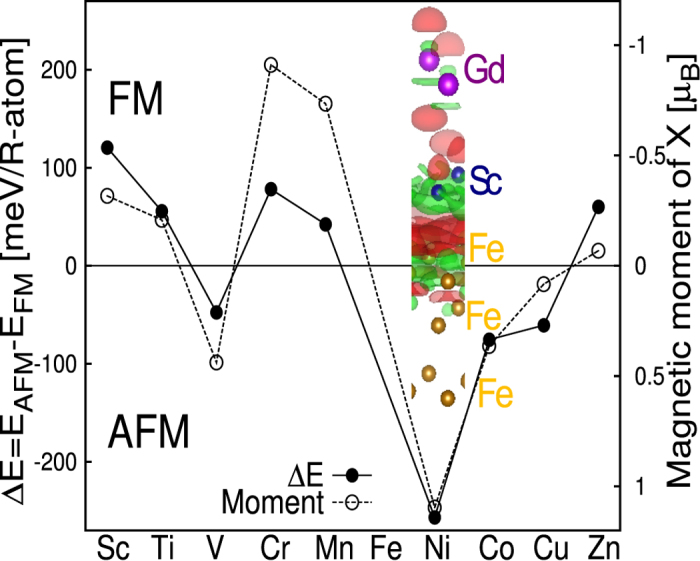
Energy differences between FM and AFM configuration and magnetic moments for different transition metals as single spacer layer. In the FM configuration, Gd and Fe are aligned parallel. In the AFM configuration, Gd and Fe are aligned antiparallel. The solid line represents the energy differences (left axis) and dashed line represents the magnetic moments (right axis). Lines are guides to the eyes. In the inset we have the charge density difference (as defined in the text) for the Fe/Sc/Gd heterostructure with 3 Fe monolayers to evidence the effect of the spacer layer. The Gd atoms are purple, the Sc atoms are blue and the Fe atoms are gold.

First, we look at the single spacer layer case scanning all the *3d* transition metals as interlayer between Fe and Gd layers. The difference in the total energy of a ferromagnetic and an antiferromagnetic ordering (*E*_*AFM*_ − *E*_*FM*_) between Fe and Gd is a measure of the interatomic magnetic exchange interaction and hence provides an estimation of the Curie temperature of the heterostructure. The results are shown in Fig. 1, where the calculated energy difference between parallel and anti-parallel configurations of Fe and Gd moments is plotted for different *3d* transition metal monolayer as a spacer between Fe and Gd. Positive value for the energy difference indicates a parallel alignment of the Fe and Gd moments to be favourable while the negative value indicates an anti-parallel alignment. The figure also shows (the right axis) the calculated induced/intrinsic magnetic moment of the spacer layer atom. The electronic configuration of the spacer layer and hence the number of *3d* electrons is crucial for the magnetic coupling apart from the induced or intrinsic magnetic moment of spacer atoms. The spacers with electronic configurations *d*^1^, *d*^2^, *d*^4^, *d*^5^, *d*^10^ lead to ferromagnetic coupling between Fe and Gd moments while the spacers with *d*^3^, *d*^6^, *d*^7^, *d*^8^, *d*^9^ electronic configuration leads to an antiferromagnetic coupling of Fe and Gd moments. It should be remarked that the electronic configurations of the ferromagnetic transition metals *d*^6^ (Fe), *d*^7^ (Co), *d*^8^ (Ni) yield an antiferromagnetic coupling as known from the previous studies^11^,^12^,^13^ on *3d*-*4f* magnetic interfaces. The value obtained for the Fe/Cr/Gd heterostructure is closer to what is reported in the paper by Sanyal *et al*.^5^. The magnetic moments of the Cr atoms for the ferromagnetic and antiferromagnetic configuration are respectively 1.2 and 0.9 *μ*_*B*_. We also tried to use Co instead of Fe in the trilayer.

The Co/Cr/Gd system compared to the Fe/Cr/Gd system reduces the ferromagnetic coupling between the transition metal and the rare earth as expected since Fe has larger critical temperature respect to the bcc Co^14^.

We analyzed the charge density difference *ρ*_*Fe*+*Sc*+*Gd*_ − *ρ*_*Fe*+*Gd*_ − *ρ*_*Sc*_ shown in the inset in Fig. 1. Positive values are indicated by red contours and negative value are indicating by green contours. We study the system with and without the Sc monolayer with the same atomic positions to check the influence of the Sc layer. The charge density of the Fe interface layer is strongly modified by the spacer layer following the ionic displacement, while the Gd charge density is almost unchanged in the presence of the spacer layer. In this case, as in general in the Fe/X/Gd heterostructure, the layers that undergo stronger modification from magnetic and structural points of view are the interface Fe and the X_1_ layers while the Gd layer is less influenced.

Now, we discuss the importance of induced/intrinsic magnetic moments of the single spacer layer for the magnetic ground state of the heterostructures. The magnetic atoms Cr, Mn, Co and Ni have intrinsic magnetic moments. The other elements, intrinsically non-magnetic, show induced magnetic moments that can reach a maximum of 0.44 *μ*_*B*_ in the case of V. The only element that shows ferrimagnetic behaviour in the plane is Mn as it presents a *G*-type antiferromagnetism with spin down moment (−2.72 *μ*_*B*_) larger than spin up moment (+1.25 *μ*_*B*_) along with a small buckling in the layer (0.01 Å). For this system, we plot the average magnetic moment of the ferrimagnetic layer in Fig. 1. All the other elements display a ferromagnetic alignment of uniform spin moments. In Fig. 1, we can clearly observe a correlation between the magnetic moments and the energy differences. Spacer layers coupling ferromagnetically with Fe tend to couple antiferromagnetically with Gd giving rise to an effective antiferromagnetic coupling between Fe and Gd. Spacer layers that couple antiferromagnetically with Fe tend to couple antiferromagnetically with Gd also, resulting in a situation with ferromagnetically coupled Fe and Gd. As a result, the total moment of the heterostructure is increased.

**Figure 2:**
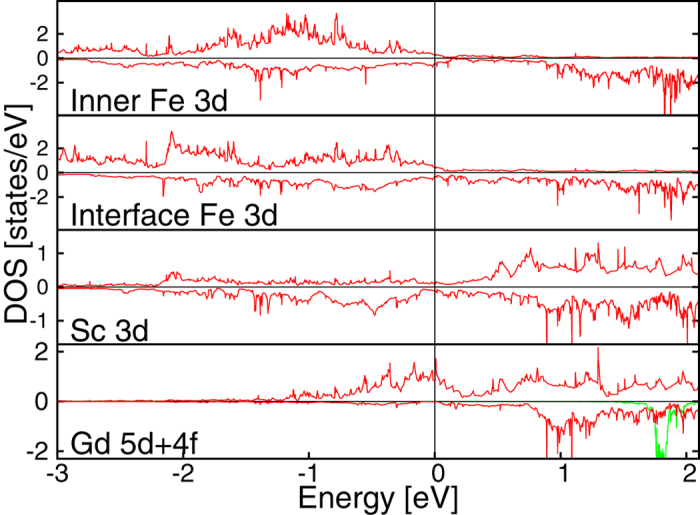
Layer projected DOS for the ferromagnetic phase of the Fe/Sc/Gd heterostructure with one Sc layer. The *3d* DOS of inner Fe layer, interface Fe layer and Sc layer are plotted in red. The *5d* and *4f* Gd layer DOS are plotted respectively in red and green. The *4f* DOS is divided by a factor 20. Majority (minority) spin is represented in the upper (lower) half of the panel.

Now, we present the electronic structure of the heterostructure. The Coulomb repulsion localizes the *4f* states of Gd. The majority *4f* states are 7 eV below the Fermi level, while the minority are 1.8 eV above the Fermi level as can be seen from the DOS plot in Fig. 2. The Gd DOS at the Fermi level is dominated by the *5d* electrons. All the layers present a metallic magnetic behavior at the Fermi level due to the *5d* electrons. We observe from the orbital projected DOS (not shown here) that a slightly larger contribution to the spacer magnetic moment comes from the t_2*g*_ electrons that have the lobes in the direction of the Fe atoms. This shows that the Fe atoms influence both magnetic and orbital properties of the spacer layer.

### Ab-initio study of multiple interlayers

**Figure 3:**
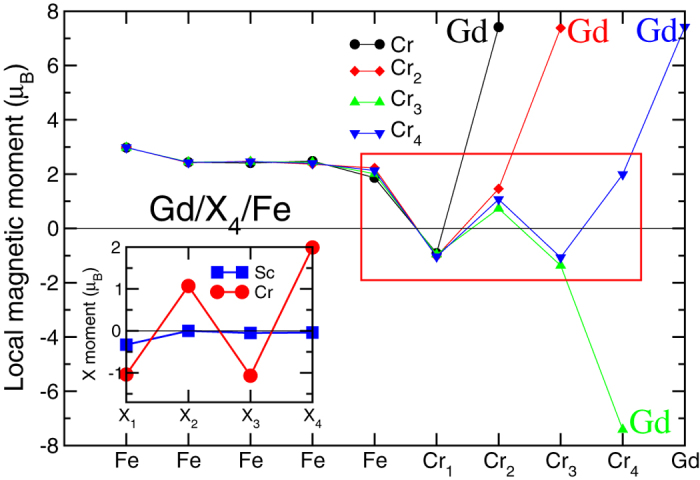
Magnetic profile of the Fe/Cr/Gd heterostructure. We plot the results for 1 (black), 2 (red), 3 (green) and 4 (blue) Cr spacer layers. The comparison with Sc is shown in the inset.

Now, we discuss the results for heterostructures with spacer layer thicker than one ML. In Fig. 3, we show layer-resolved magnetic moments for Fe/Cr/Gd heterostructures with up to 4 Cr spacer layers. Also, a comparison has been made with Fe/Sc/Gd heterostructures with up to 4 Sc layers as the spacer. Several interesting observations can be made. The Cr layer nearest to Gd couples antiferromagnetically with it in the case of one spacer layer whereas it couples ferromagnetically in all other cases. This is because the Fe layer strongly influences its nearest neighbor Cr layer and vice versa. Also, the magnetic moment is different between the two cases. The Cr layer closest to Fe has a magnetic moment around 1.0 *μ*_*B*_ but the one nearest to Gd has always a larger magnetic moment reaching 2.0 *μ*_*B*_ when there are 4 Cr monolayers. This is due to the complex interplay between the electronic structures of Cr and the interface Fe and Gd layers. The magnetic moment of the interface Fe layer is reduced compared to its bulk value while there are no substantial changes in the magnetic properties of Gd for different cases.

**Figure 4:**
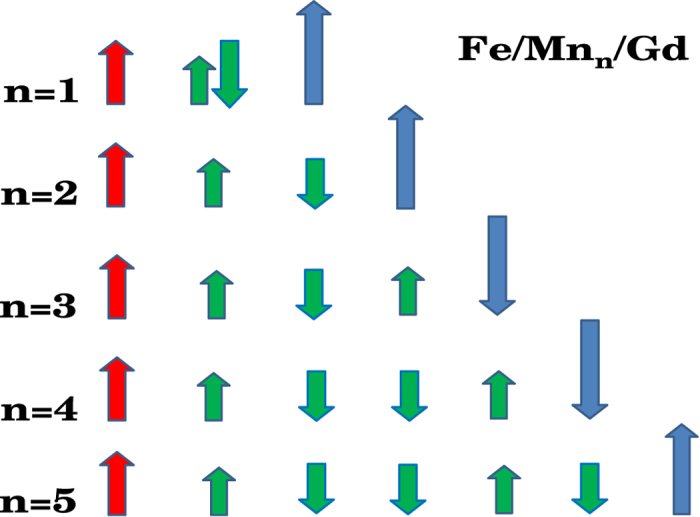
Magnetic configuration of the Fe/Mn_*n*_/Gd heterostructure for *n* = 1–5 ML. We just show the spin of the interface Fe layer. The Fe spins are red, the Mn spins are green and the Gd spins are blue.

Differently from the other spacer layers, our calculations indicate a complex arrangement of the Mn spin moments as function of the number of Mn MLs, n, for the Fe/Mn_*n*_/Gd trilayer. With an increase in the number of ML, *n*, the ferrimagnetic order of Mn disappears and the ferromagnetism in the plane of the layers is observed. However, the ferromagnetic Mn layers do not always couple antiferromagnetically between them as in Cr. We report in Fig. 4 the magnetic configurations of the Fe/Mn_*n*_/Gd heterostructures with *n* ML of Mn. The ferrimagnetic order is the ground state for the *n* = 1 case, while for larger number of monolayers, the system adopts a layer-by-layer antiferromagnetic ordering. This magnetic ordering was already found with just the Fe substrate^15^. For *n* ≥ 2 ML, the corresponding interface Mn layers always couple ferromagnetically with Fe and antiferromagnetically with Gd. As a consequence of the different magnetic orderings between the Mn layers, we observe a ferromagnetic coupling between Fe and Gd for *n* = 1, 2, and 5 ML, while we observe an antiferromagnetic coupling for *n* = 3, 4 ML.

**Figure 5:**
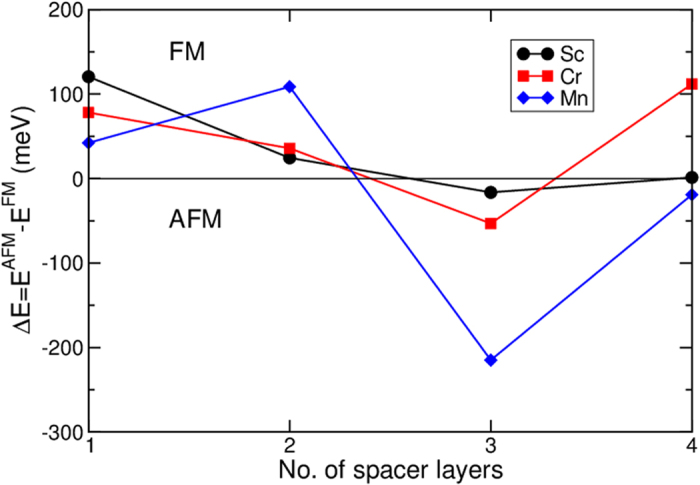
Energy differences between FM and AFM configuration as a function of the number of spacer layers. In the FM configuration, Gd and Fe are aligned parallel. In the AFM configuration, Gd and Fe are aligned antiparallel. We plot the results for Cr (red), Mn (blue) and Sc (black). The horizontal dashed line is the level of the antiferromagnetic interlayer, none of the non-magnetic materials can reach it for more than 1 layer. Lines are guides to the eyes.

Studying the non-magnetic elements, we can observe that the magnetic moment is induced mainly from the Fe layer as we see from the inset in Fig. 3. We observe a relatively large induced magnetic moment of 0.3 *μ*_*B*_ on the interface Sc_1_ that is suppressed for the other layers. The Fe/Sc/Gd heterostructure presents an oscillating behavior in the coupling between Fe and Gd in Fig. 5. This oscillatory behavior was found also in the other non-magnetic transition metals^16^,^17^. The magnetization in the Fe interface layer is reduced as commonly found in FM/NM interface^5^,^6^. In the case of the Fe/Sc/Gd system, the reduction of the magnetization in the Fe interface layer is around 20%.

Now, we present the results of the range of exchange coupling between Gd and Fe across the spacer layers in Fig. 5. Among the non-magnetic spacer layers, Sc is the most promising as it offers a substantial exchange coupling between Fe and Gd for one monolayer spacing. However, the exchange coupling decays rapidly with the spacer thickness as was shown in similar systems for non-magnetic spacer^18^. As a decaying behaviour is found in all tested systems, we assume that beyond one monolayer the best materials to ferromagnetically couple the transition metals and rare earth are the antiferromagnetic materials Cr and Mn. For the two antiferromagnetic elements and for Sc we also analyze the heterostructure system up to four layers as shown in Fig. 5. For the non-magnetic spacers, we observe an oscillation of the magnetic coupling thanks to the induced magnetic moment as we can observe for the Sc in Fig. 5. In case of Sc spacer when we reach four layers the magnetic coupling disappears, while in Cr and Mn the oscillating behaviour is sustained by the intrinsic magnetic moment as experimentally observed for Cr spacer^19^ and Fe/Cr/Gd/Gr superlattices^20^. For the Fe/Cr/Gd/Cr superlattice, a RKKY type of behavior was found in contrast to the non-magnetic interlayers.

One should note that in all these calculations, we have neglected the interdiffusion of atoms across the interface by considering an ideal interface. The qualitative correspondence between the ideal interface and the real case is always possible. For instance, we have checked the case of the diffusion of the Fe atoms in the spacer layer Fe/Cr/Gd studying a system Fe/Cr_0.75_Fe_0.25_/Gd. In this case the direct coupling between Fe and Gd, without the X layer, is antiferromagnetic destroying the ferromagnetic coupling between the Fe and Gd layers mediated by the Cr atoms.

### Experimental magnetic measurements with non-magnetic Sc interlayer

**Figure 6:**
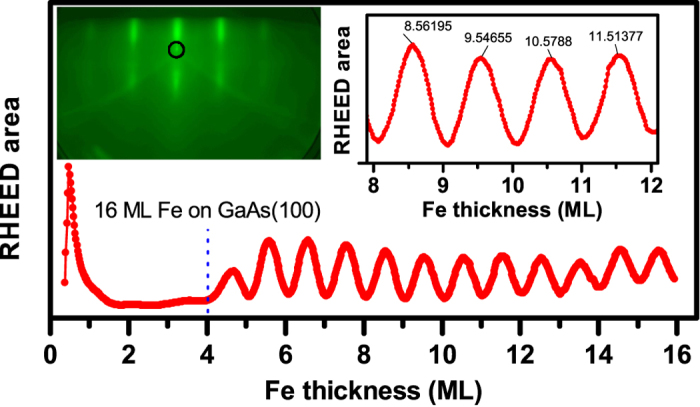
RHEED intensity and pattern. The figure shows the RHEED intensity, integrated over the area marked in the left inset, verses the thickness (in monolayers) of Fe layer determined from quartz balance. The RHEED pattern indicates an island growth of Fe up to 4 ML and a layer-by-layer growth after that.

The above theoretical results are compared with two molecular beam epitaxy (MBE) grown trilayer systems GaAs(100)/Fe_15_/Sc_*n*_/Gd_30_ and GaAs(100)/Fe_15_/Mn_*n*_/Gd_30_ (subscripts denote the number of monolayers of each layer); one with a non-magnetic, Sc, spacer and the other with a magnetic, Mn, spacer. The reflection high energy electron diffraction (RHEED) pattern collected on the GaAs after sputter cleaning confirms earlier reports of 4 × 6 surface reconstruction in GaAs^21^. A typical RHEED intensity oscillations for the growth of Fe on GaAs(100) are shown in Fig. 6. It is clear from these oscillations that Fe initially grows in 3-dimensions up to 4th ML and then it follows a layer by layer growth resulting in well-defined intensity oscillations corresponding to full and half monolayer coating as demonstrated in the right inset of the Fig. 6. Similar RHEED analysis has indicated that the Mn on Fe grows layer by layer right from the 1st ML and that the Sc also grows layer by layer on Fe.

**Figure 7:**
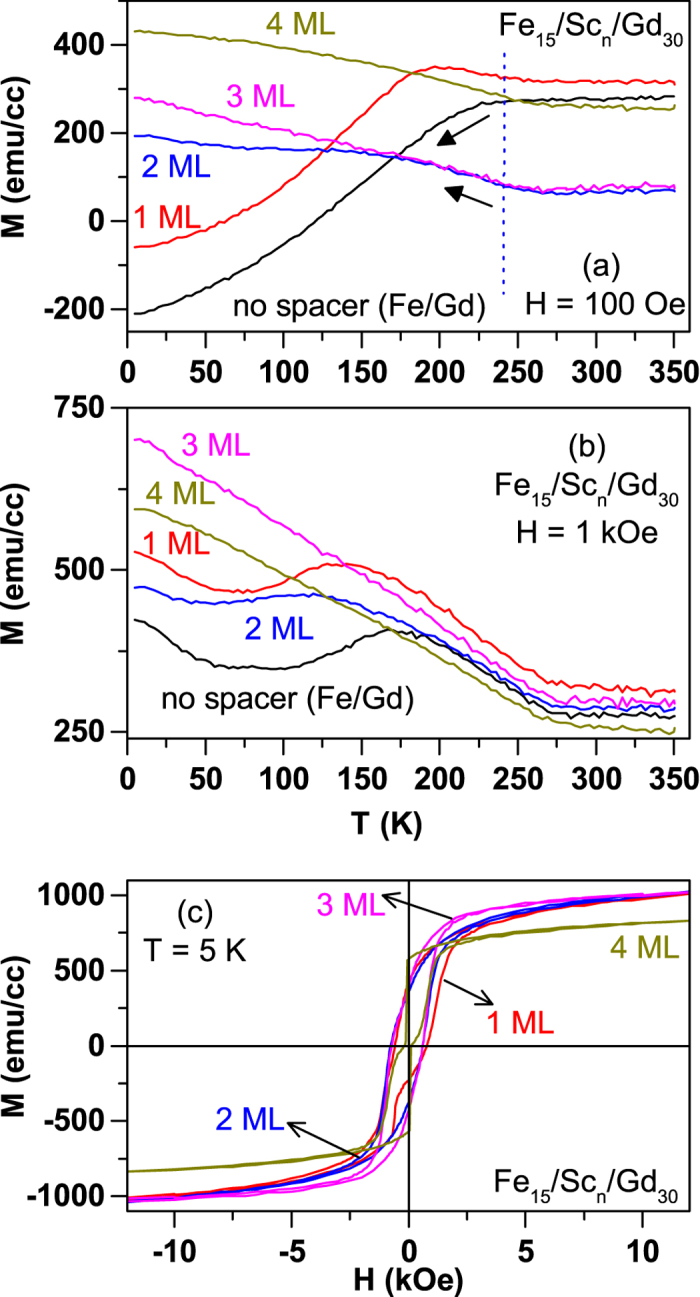
Magnetization data measured on Fe_15_/Sc_*n*_/Gd_30_ trilayer samples. The field-cooled (FC) magnetization data measured on GaAs(100)/Fe_15_/Sc_*n*_/Gd_30_ trilayer samples are shown for two different measuring fields of (**a**) 100 Oe and (**b**) 1 kOe. The data for the reference sample (Fe/Gd) are also shown as indicated.

Figure 7 shows the field cooled (FC) magnetization, with H = 100 Oe and 1 kOe, versus temperature data for the samples with Sc spacers along with the reference sample of GaAs(100)/Fe_15_/Gd_30_ for comparison. The reference sample Fe/Gd and the samples with 1ML Sc spacer show qualitatively similar behaviour in that the FC magnetization gradually decreases with lowering of the temperature below ~250 K, when measured in 100 Oe field. It is important to note that the decrease in magnetization starts below 250 K, which coincides with the ordering temperature of 30 ML Gd sample (data not shown). This decrease in FC moment on cooling is a clear indication of antiparallel alignment of Fe and Gd moments. On the other hand, the trilayers with Sc spacer thickness ≥2 ML display qualitatively opposite behaviour where the FC magnetization starts to increase with lowering temperature and the onset of increase once again coincides with the ordering temperature for 30 ML Gd. So, on comparing the data of the reference Fe/Gd bilayer with the trilayer samples of Fe/Sc/Gd one can conclude that the antiferromagnetic coupling between Fe and Gd moments is changed to a ferromagnetic coupling by inserting 2 monolayers of Sc. This is in qualitative agreement with the theoretical results reported in Fig. 5. However, the current macroscopic magnetic measurements cannot give any idea about the number of Gd layers coupled to Fe and if there has been an enhancement of Gd ordering temperature as a result of this coupling.

The main discrepancy between the experiment and theory is the persistence of antiferromagnetic coupling between Fe and Gd moments in the sample with 1 monolayer Sc spacer. We believe that this is due to the experimental difficulties in achieving a clean interface. We suspect that the interface alloying of Fe and Sc layers occurs in the order of one monolayer. Therefore, the diffused Fe atoms in the Sc monolayer couple antiferromagnetically the Fe and Gd side of the heterostructure as theoretically observed for Fe/Cr_0.75_Fe_0.25_/Gd heterostructure discussed earlier. The FC magnetic data shown in panel (b) of Fig. 7 indicates the weakness of antiferromagnetic coupling between Fe and Gd. By increasing the measuring field to 1 kOe, the magnetization curves for the reference Fe/Gd sample and for the sample with 1 ML Sc spacer, both with intrinsic antiferromagnetic coupling of Fe and Gd, are strongly effected.

To further understand the features in 1 kOe FC data, we have measured the magnetic hysteresis loops of these trilayer samples at 5 K. This data, shown in Fig. 7(c), indicates subtle changes in the magnetic anisotropy between the samples with different Sc spacer layers. The sample Fe_15_/Sc_4_/Gd_30_ shows two switching fields, presumably related to Fe and Gd layers, confirming the weak coupling between Fe and Gd for this Sc thickness. In addition, the saturation magnetization for the Fe_15_/Sc_4_/Gd_30_ sample is strikingly smaller compared to other samples with a Sc spacer. This may be due to the dipolar field effects that arise when the Fe and Gd layers are suitably separated. This also explains the fact that the FC magnetization, in 1 kOe field, for Fe_15_/Sc_4_/Gd_30_ sample is lower than that of Fe_15_/Sc_3_/Gd_30_.

### Experimental magnetic measurements with magnetic Mn interlayer

**Figure 8:**
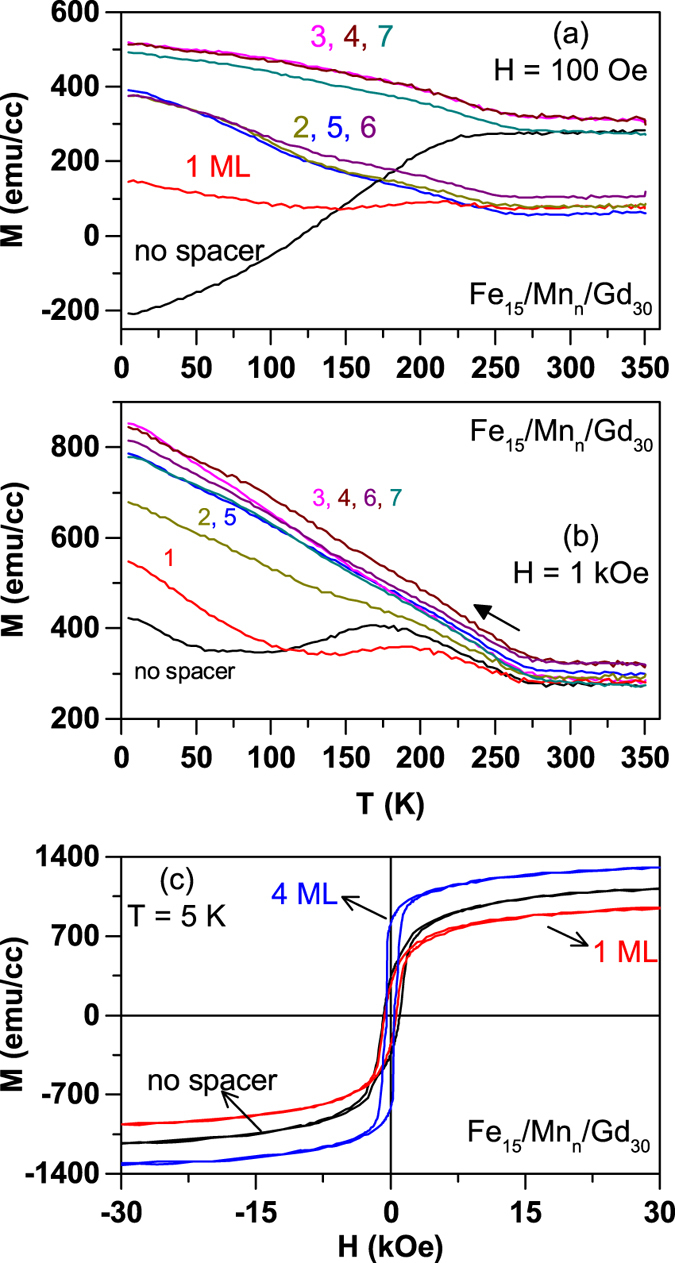
Magnetization data measured on Fe_15_/Mn_*n*_/Gd_30_ trilayer samples. The field-cooled (FC) magnetization data measured on GaAs(100)/Fe_15_/Mn_*n*_/Gd_30_ trilayer samples are shown for two different measuring fields of (**a**) 100 Oe and (**b**) 1 kOe. The data for the reference sample (Fe/Gd) are also shown as indicated. An apparent grouping of magnetic data for samples with *n* = 2, 5, 6 ML Mn and *n* = 3, 4, 7 ML Mn spacer is worth noting.

In Fig. 8, we show the FC magnetization - temperature curves for the trilayers with Mn spacers along with the reference Fe/Gd bilayer for comparison. The data for the trilayer with 1 ML Mn spacer does not show similarity with the reference sample, however, it does neither indicate a ferromagnetic coupling between Fe and Gd. The trilayers with Mn spacers of thickness *n* ≥ 2 ML indicate a clear gradual raise in the FC magnetization on cooling below ~250 K, indicating a parallel alignment of the Gd moment with that of the Fe. A point worth noting in the panel (a) is the grouping of the magnetization curves depending on the number of Mn spacer layers. The samples with *n* = 2, 5, and 6 ML Mn show similar magnetic behaviour while the samples with *n* = 3, 4 and 7 ML Mn show a different magnetic behaviour than the first group. This is in good qualitative agreement with the theoretical results reported in Fig. 4.

The magnetization curves measured in a higher field of 1 kOe [Fig. 8(b)] show that the trilayer with 1 ML Mn spacer is slightly effected while the other samples with thicker Mn spacers do not display the grouping behaviour that is observed while measuring in 100 Oe field. The magnetic hysteresis loops measured at 5 K for these samples are shown in Fig. 8(c) and these data show that the coupling between Fe and Gd persists up to higher Mn thickness and also leads to a higher saturation magnetization compared to that of the trilayer systems with Sc spacer. It is also important to note that there can be two different magnetic layers within Gd as suggested in the case of Fe/Cr/Gd trilayers reported in ref. 5. The authors of ref. 5 predicted that only a few ML of Gd are coupled to the Fe via Cr while the remaining Gd layers retain their intrinsic magnetic ordering temperature. Therefore, one must interpret the present experimental results as resulting from different magnetic contributions of Gd layers coupled with the Fe/Gd magnetic coupling. Nevertheless, it is clear from the present experimental results, which compare the trilayer samples with that of the reference Fe/Gd sample, that it is possible to break the antiferromagnetic coupling between Fe and Gd and align their moments in parallel by having a third material (Sc, Mn) as a spacer layer. Synchrotron based element resolved magnetic measurements will be performed on these trilayer structures and the results will be published elsewhere along with the more detailed description of the growth and structure of the experimental samples.

## Conclusions

In conclusion, by means of first-principles density functional theory, we determined the structural, electronic and magnetic properties of Fe/X/Gd heterostructures with X being *3d* transition metal as the spacer between Fe and Gd. Two trilayer systems Fe/Sc/Gd and Fe/Mn/Gd have also been studied experimentally. Depending on the nature of the magnetic coupling, we may have three different transition metal categories: the non magnetic, the ferromagnetic and the antiferromagnetic elements. Co and Ni couple ferromagnetically to Fe and antiferromagnetically to Gd. Non magnetic early transition metal elements as Sc can create a strong ferromagnetic coupling for one interlayer but when we increase the number of layers we observe an oscillating behavior with a rapid decay of the magnetic coupling. Antiferromagnetic Cr and Mn are the only elements able to produce a ferromagnetic coupling for a long range. The experimental data on Fe/Sc/Gd and Fe/Mn/Gd confirm the possibility to align the Fe and Gd moments parallel, albeit with some remaining challenges arising from the interface roughness and some structural mismatch between Gd and the spacer layer material. We hope that this systematic study of the magnetic coupling between high critical temperature and high spin magnetic moment ferromagnets may open the route for the realization of device based on transition metals and rare earth superlattices.

## Methods

### Experimental details

We have used molecular beam epitaxy (MBE) technique to prepare trilayer films of GaAs(100)/Fe_15_/Sc_*n*_/Gd_30_ with *n* = 1–4 ML and GaAs(100)/Fe_15_/Mn_*n*_/Gd_30_ with *n* = 1–7 ML. A reference sample of GaAs(100)/Fe_15_/Gd_30_ is also prepared for comparison of magnetic properties. In a typical growth, the GaAs (100) substrate is rinsed with acetone and isopropanol followed by drying, blow drying with nitrogen gas. It is then inserted into a ultra high vacuum (UHV) chamber with a pressure lower than 6 × 10^−10^ mbar. The substrate is then heated to ~600 °C and cleaned by sputtering at 3 kV, 25 mA for 1 hour in an Ar pressure of 5 × 10^−5^ mbar. Auger spectra collected before and after sputtering are used to confirm that the substrate is clean from surface impurities. The reflection high energy electron diffraction (RHEED) is used to confirm the smooth GaAs surface. The Fe (at 0.03 Å/s), Sc or Mn (at 0.02 Å/s) and Gd (at 0.03 Å/s) layers are grown on the clean GaAs substrate with the growth rate monitored by quartz crystal balance and the growth surface is monitored by recording RHEED pattern with a 3 sec interval. In the final step, all the films were capped with a 2 nm Cr layer to prevent oxidation. A Quantum Design Dynacool PPMS is used for the magnetic characterization of these trilayer samples.

### Computational details

We have performed first-principles density functional calculations by using the VASP^22^ package based on plane wave basis set and projector augmented wave method^23^. A plane-wave energy cut-off of 330 eV has been used. For the treatment of exchange-correlation, Perdew-Burke-Ernzerhof 24 generalized gradient approximation has been considered. In order to include strong electron correlations, we have considered a Hubbard *U* approach^25^, commonly used to describe the electronic structures of Gd to avoid *4f* electrons near the Fermi level. The *U* value of 5 eV for Gd-*4f* orbitals has been considered following the recommendations in published articles. The use of a different set of parameters as *U* = 6 eV and *J*_*H*_ = 1 eV does not modify the results.

In our simulations, the heterostructures were constructed using a lateral supercell 2*a* × 2*a* with the lattice parameters of bulk Fe. For the farthest layers from the interface the value of interplanar separation for bulk Fe (~1.43 Å) is attained. We always used 5 Fe layers and 1 Gd layer in the Fe/X/Gd heterostructure. The X layers between Fe and Gd are termed as “spacer layers” or “interlayers” for which the bcc symmetry imposed by the Fe substrate has been considered. The closest Fe layer to the interface is termed interface Fe layer. In this heterostructure, the interface Fe layer has two inequivalent Fe atoms. The first is the Fe atom with the same in-plane position of the Gd and it is the closest Fe atom to the Gd. Instead, the second Fe atom corresponds to an empty site in the Gd layer. These two inequivalent Fe atoms have different magnetic moments and positions along the z-axis producing a layer buckling. Indeed the first Fe atom goes towards the Fe bulk pushed by the Gd and the buckling is of the order of 0.02 Å for different spacer layers. The difference in the magnetic moments is always less than 0.1 *μ*_*B*_ and in this paper we just report the average magnetization of the plane. The other Fe layers have all equivalent atoms. A 6 × 6 × 1 *k*-point set was used for Brillouin zone integrations in the Monkhorst-Pack scheme. A 8 × 8 × 1 *k*-point mesh was used for the calculations of density of states (DOS). The geometries were relaxed until the forces on all atoms were reduced to 20 meV/Å.

## Additional Information

**How to cite this article**: Autieri, C. *et al*. Recipe for High Moment Materials with Rare-earth *3d* Transition Metal Composites. *Sci. Rep.* **6**, 29307; doi: 10.1038/srep29307 (2016).
